# The Efficacy and Safety of Botulinum Toxin A for the Treatment of Rosacea: A Systematic Review

**DOI:** 10.7759/cureus.51304

**Published:** 2023-12-29

**Authors:** Ahmed A Alsaati, Dalal Alsaadoun, Lina I Kinkar, Riam Saleh Alkhamis, Walaa Abdu Ahmed, Alhanouf Hassan Almathami

**Affiliations:** 1 Dermatology, College of Medicine, King Faisal University, Al-Hofuf, SAU; 2 Medicine and Surgery, College of Medicine, Umm Al-Qura University, Makkah, SAU; 3 Dermatology, King Saud Medical City, Riyadh, SAU; 4 Medicine and Surgery, College of Medicine, King Khalid University, Abha, SAU

**Keywords:** rosacea, quality of life, flushing, erythema, botulinum toxin

## Abstract

The off-label use of botulinum toxin type-A (BoNT-A) in treating rosacea seems encouraging, but the evidence is still lacking regarding its efficacy and safety. This study was conducted to summarize the evidence regarding the efficacy and safety of BoNT-A in the treatment of rosacea patients. A comprehensive literature search was conducted in several databases, and 17 studies were included. Before-after and split-face comparisons showed that BoNT-A significantly alleviated the symptoms of facial erythema and flushing and improved the patient’s quality of life/satisfaction. However, the symptoms recurred three to six months post-injection, requiring repeated treatments in some patients. The pooled rates of post-injection localized erythema, ecchymosis, and facial muscle affection represented 24.6%, 5.1%, and 4.3%, respectively. BoNT-A seems to be effective in alleviating the symptoms of rosacea with a low rate of adverse events. However, the recurrence of the symptoms a few months after the injection requires repeated sessions, which may raise cost-effectiveness issues. Large-scale clinical trials are required to confirm the effectiveness and define the optimal dosing regimen and the rate of recurrence. Future studies should allow for an adequate follow-up after the treatment, with repeated measurements of the outcomes.

## Introduction and background

Rosacea is a chronic inflammatory disease that is characterized by repeated episodes of cutaneous facial manifestations, including flushing, persistent erythema, telangiectasia, papules and/or pustules, and phymatous changes [1,2]. Some patients may also suffer from skin itching or burning [3].

The pathogenesis of rosacea is complex and involves a variety of factors that can trigger both inflammatory and vascular responses. Several factors may contribute to its pathogenesis. In addition to genetic factors, other triggering factors such as microbial elements (including demodex), ultraviolet exposure, diet, neurovascular factors, and stress, as well as immune dysregulation, have been implicated in rosacea [4-6].

Rosacea usually first appears between 30 and 50 years old but it can start at any age [7,8]. A recent systematic review estimated the global prevalence of rosacea to be 5.5% among the adult population, with nearly equal rates in men and women [9]. However, older studies reported female gender predilection [7,8]. Traditionally, four subtypes are described including erythematotelangiectatic (ERT), papulopustular, phymatous, and ocular rosacea [10]. The diagnosis of rosacea depends on the presence of either fixed centrofacial erythema or phymatous changes [7]. If either of these features is absent, the diagnosis depends on the presence of two or more of rosacea’s major features, which include flushing/transient centrofacial erythema, inflammatory papules and pustules, telangiectasia (excluding alar involvement), and ocular manifestations (lid margin telangiectasia, blepharitis, keratitis, conjunctivitis, or sclerokeratitis) [1].

The clinical guidelines and expert consensus have indicated several lines for rosacea treatment [11-14], including topical agents such as brimonidine, oxymetazoline, ivermectin, metronidazole, and azelaic acid as well as oral agents such as doxycycline. In addition, laser and light-based therapies can improve telangiectasia, erythema, and phymatous changes. In some advanced cases, phyma may need surgical correction [15]. Despite the presence of various treatment options, managing rosacea remains a challenge, particularly in refractory or recalcitrant cases [2].

Recently, Clostridium botulinum toxin type-A (BoNT-A) has been introduced into the management of various dermatological inflammatory conditions [16]. Botulinum toxin type-A shows promise as a potential treatment method. Case reports and case series have reported on the efficacy of intradermal BoNT-A in improving flushing and telangiectasia in rosacea patients [17-21]. However, the use of BoNT-A in rosacea is still off-label, and the current clinical guidelines do not recommend its routine use. Therefore, the present systematic review was carried out to summarize the evidence regarding the efficacy and safety of BoNT-A in the treatment of patients with rosacea.

## Review

Methods

Methodology

The protocol of this systematic review was registered at the International Prospective Register of Systematic Reviews (PROSPERO) (registration number CRD42023423316) on 16-5-2023.

The conduction and reporting of this systematic review followed the principles of the Cochrane Handbook for Systematic Reviews of Interventions, version 6 and the Preferred Reporting Items for Systematic Reviews and Meta-Analyses (PRISMA) guidelines [22].

Research Question

Is intradermal injection of BoNT-A an effective and safe treatment for rosacea?

Research Aim and Objectives

This systematic review aimed to assess the efficacy and safety of BoNT-A in the treatment of patients with rosacea. The studied objectives included: (a) Assessment of the improvement in the symptoms of rosacea (particularly flushing and erythema) after the injection of BoNT-A and (b) Assessment of the reported adverse effects that are related to the use of BoNT-A.

Eligibility Criteria

This systematic review included observational (case reports or series) studies and clinical trials. The literature search was limited to studies published in English from inception to the 9th of April 2023. Eligible studies included patients with rosacea and included intradermal injection of BoNT-A. Studies were excluded if conducted on animals, or if flushing and injection were in a body part other than the face. We also excluded conference abstracts, duplicate records, protocols, reviews, and clinical guidelines.

Search Strategy

The literature search was conducted on the electronic databases of MEDLINE/PubMed, Cochrane Central Register of Controlled Trials (CENTRAL), EBSCO Academic Search Complete Database, Web of Science, ProQuest, and Scopus. The search included all published articles from inception till the 9th of April 2023. The search was carried out from the 1st of April 2023 to the 9th of April 2023. The used search terms included "botulinum toxin" AND "rosacea". The used search terms and the number of search results for each database are outlined in Table 1.

**Table 1:** The Search Strategy in Different Databases

Database	Search keywords	Filters	Number of results
PubMed	("Botulinum Toxins"[Mesh]) AND "Rosacea"[Mesh]	No filters	13
Cochrane library	("Botulinum Toxins"[Mesh]) AND "Rosacea"[Mesh]	No filters	204
Web of Science	(ALL=(Botulinum Toxins)) AND ALL=(Rosacea)	No filters	36
Scopus	(ALL=(Botulinum Toxins)) AND ALL=(Rosacea)	No filters	369
EBSCO	(ALL=(Botulinum Toxins)) AND ALL=(Rosacea)	No filters	15
ProQuest	(ALL=(Botulinum Toxins)) AND ALL=(Rosacea)	No filters	102

We searched the reference lists of the records obtained by electronic search aiming to find other potentially related studies.

Selection of Studies

We carried out the literature search, the screening of the titles and abstracts, the retrieval of the full text of potentially relevant records, and the assessment of each study’s eligibility for inclusion in this systematic review. The search and selection processes were checked, and any disagreements were settled by discussion.

Data Extraction

We extracted data from the included studies using a standardised data sheet. The extracted data included: (a) the characteristics of the study (the country, study design, sample size, its inclusion and exclusion criteria, and the duration of follow-up); (b) patients’ characteristics (age at the time of study and sex); (c) the disease characteristics (subtype and duration); (d) the intervention (type of toxin, dilution, and the number of sessions); (e) the improvement in symptoms and the duration of improvement or time till relapse; and (f) the adverse effects. We checked the extracted data to assess the consistency and clarity of the recording, and any disagreements were settled by discussion.

Measured Outcomes

The primary outcome was the improvement in the symptoms of rosacea (flushing, erythema, and telangiectasia). Secondary outcomes included the intervention-related adverse events, relapse rate, and time to relapse.

Assessment of the Risk of Bias in the Included Studies

We used the National Institutes of Health (NIH) quality assessment tool for the before-after (Pre-Post) study with no control group [23]. For case reports, we used the Joanna Briggs Institute (JBI) Critical Appraisal Checklist for Case Reports [24].

Data Synthesis

A narrative synthesis table was created for each outcome to summarize the used methods for assessing the outcome and the individual study’s main findings. For pooling the incidence rates of the adverse events, we used the Metaxl add-in of Microsoft Excel (version 5.3, Epigear International, www.epigear.com). The Cochran Chi-square heterogeneity test and the I2 index were performed to assess the heterogeneity among the studies. Significant heterogeneity was defined as a Cochran Chi-square test yielding a p-value<0.1 or the I2 index being 50% or above. The random-effects model was used to pool the incidence rates if heterogeneity was significant, while a fixed-effect model was employed for non-significant heterogeneity. Forest plots were drawn for the incidence rates of adverse events.

Results

Results of Literature Search and Study Selection

The search strategy of online databases yielded 539 records, out of which 188 records were excluded (187 were duplicates and one article was not published in English). Next, screening the titles and abstracts of the remaining 351 records resulted in the exclusion of 337 records due to the publication type (n = 62), non-relevance (n = 270), and conduction on animals (n = 4) or in vitro (n = 1).

**Figure 1:**
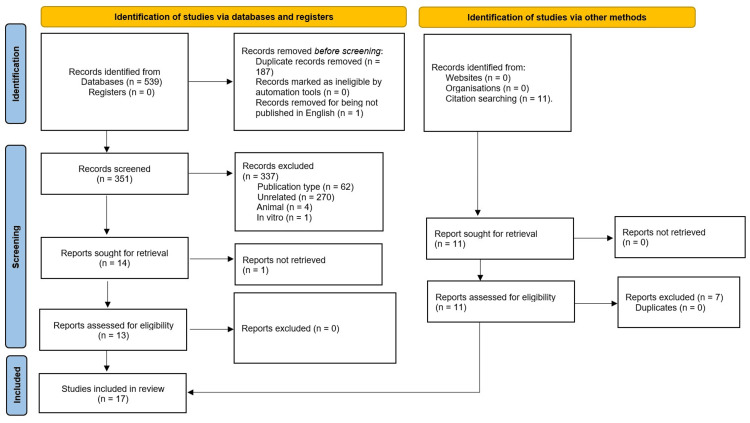
The Preferred Reporting Items for Systematic Reviews and Meta-Analyses (PRISMA) Flow Chart for the Results of the Literature Search and Study Selection

The full texts of the remaining 14 records were all retrieved except for one study. All the retrieved 13 full-text records were eligible to be included in this systematic review. On searching the reference lists of retrieved full texts, we found 11 potentially relevant records from which seven were excluded as the diagnosis was not ascertained to be rosacea. Finally, 17 studies were included in this systematic review (Figure 1).

Basic Characteristics of the Included Studies

Four studies were clinical trials [25-28]; four studies were prospective pilot [21,29-31], four were case series [32-35]; and five studies were case reports [17,19,36-38]. The studies were conducted in the USA [17,21,36], Korea [19,26,31], Iran [25], Brazil [30,34], Israel [33], Chile [27], Columbia [38], Italy [32], and China [28,35]. One study was conducted in different centres in the United Kingdom, Denmark, and Russia [29] and one study did not mention the country [37]. The sample size varied widely from one to three patients in case reports and from six to 23 in other studies. Most patients were females. The most prevalent subtype of rosacea was erythematotelangiectatic, with some studies including as well papulopustular rosacea. The follow-up duration varied from as short as one month after the intervention [25] up to nine months [29] (Table 2).

**Table 2:** Characteristics of the Included Studies (N = 17) ERT: erythematotelangiectatic; NR: not recorded; CT: clinical trial

Study	Study design	Country	Sample size	Age (years)	Sex (F: M)	Disease subtype	Follow-up (months)
Dayan [17]	Case report	USA	2	50 & 59	F	ERT	3
Bloom [21]	Prospective pilot	USA	15	Mean: 54	80% F	ERT (mild-to-moderate)	3
Park [19]	Case report	Korea	2	36 & 49	F	ERT	3
Eshghi [25]	CT	Iran	6	NR	F	NR	1
Bharti [37]	Case report	NR	1	NR	M	ERT & Papulopustular	NR
Silva [34]	Case series	Brazil	6	Range: 20-70	F	ERT	6
Park [31]	Prospective pilot	Korea	20	Mean: 35.95±11.56	18 F: 2 M	ERT	2
Friedman [33]	Case series	Israel	16	Mean: 41 (range: 23–45)	F	ERT & Papulopustular	6
Kim [26]	CT split face	Korea	23	Mean: 35.26±10.9 (range: 21–49)	17 F: 6 M	ERT (mild to moderate)	3
Al-Niaimi [29]	Prospective pilot	UK, Denmark, Russia	20	NR	NR	ERT (moderate-to-severe)	9
Gaón [27]	CT split face	Chile	18	Mean: 41 (range: 24-68)	17 F: 1 M	ERT (mild to moderate) or Papulopustular	3
Vasconcellos [30]	Prospective pilot	Brazil	10	Range: 19-60	8 F: 2M	NR	3
Luque [38]	Case reports	Colombia	3	Range: 28-39	2 F: 1 M	ERT & Papulopustular	1
Babadjouni [36]	Case report	USA	1	49	F	Papulopustular	4
Calvisi [32]	Case series	Italy	15	Mean: 47 (range: 35-57)	NR	ERT	4
Tong [28]	CT split face	China	22	Range: 20-39	F	ERT	6
Yang [35]	Case series	China	16	Mean: 30±13	F	ERT	6

The used types of botulinum toxin type-A included onabotulinumtoxinA [17,19,27,29-32], abobotulinumtoxinA [21,29,33,38], incobotulinumtoxinA [38], and prabotulinumtoxinA [26]. Six studies did not specify the type of BoNT-A [25,28,34-37]. Intradermal injection of BoNT-A was done in all studies, but one study used a novel non-laser thermal resurfacing system [33], and another study used electroporation [27], in order to increase the delivery of BoNT-A. Six studies provided more than one session of treatment, either as a part of their original regimen [17,28,29,33] or due to the recurrence of the rosacea symptoms [17,35,37] (Table 3).

**Table 3:** Intervention in the Included Studies (N = 17) BoNT-A: botulinum toxin type A; NR: not recorded

Study	Toxin type	Dilution	Dosing	Repeated sessions	Co-treatment
Dayan [17]	onabotulinumtoxinA	7 cc of saline solution per 100 units.	Multiple intradermal microdroplet injections of BoNT-A (8 to 12 U per affected cheek area), with 0.5 cm spacing.	Repeated after two weeks and four months.	Intense pulsed light (some cases).
Bloom [21]	abobotulinumtoxinA	300 U of BoNT-A was reconstituted with 3 mL sterile 0.9% saline.	Intradermal injection of 15 to 45 units.	NR	NR
Park [19]	onabotulinumtoxinA	50 U of BoNT-A was reconstituted with 2.5 ml sterile saline to achieve a concentration of 2 U/0.1 ml.	In session one: 15 U in each cheek and 3 U in each chin and the supra-eyebrow area. In session two (one week later): 5 U in each cheek and 2 U in each chin and the supra-eyebrow area. Injections were 1 cm apart.	NR	NR
Eshghi [25]	NR	NR	1 U of BoNT-A was injected intracutaneously in every square cm (total dose: 30 U per session on both sides of cheeks).	NR	NR
Bharti [37]	NR	BoNT-A diluted to 10 units/ml.	0.05 ml microdroplet intradermal injections with 0.5 cm spacing under topical anaesthesia.	Repeat once every four to five months	NR
Silva [34]	NR	100 U of BoNT-A were diluted in 5 ml 0.9% of saline solution (2 U/0.1 ml).	0.2 to 0.5 U were intradermally injected per point, with 0.5 cm spacing. Total dose: 12 to 30 U.	NR	NR
Park [31]	onabotulinumtoxin A	50 U of BoNT-A was reconstituted with 2.5 ml of sterile saline to achieve a concentration of 2 U/0.1 ml.	A total of 20 units of BoNT-A was used for each patient, with 1 cm spacing.	NR	No
Friedman [33]	abobotulinumtoxinA	100 U of BoNT-A in 3 ml of bacteriostatic saline.	Topical application assisted by ultrasound impact system.	Two treatment sessions with one-month interval	Novel thermomechano-ablative device (Tixel; Novoxel, Israel) and topical Trolamine, broad-spectrum sunscreen with SPF 50.
Kim [26]	prabotulinumtoxinA	Diluted with injectable NS to a concentration of 1 U per 0.1 mL	A total of 15 U of BoNT-A was injected intradermally into one cheek, while the other cheek was injected with the placebo (NS), with 1 cm intervals.	NR	no
Al-Niaimi [29]	abobotulinumtoxinA onabotulinumtoxin A	abobotulinum: 500 U in 5 mL, onabotulinum: 100 U in 2.5 mL	abobotulinum: 20 to 50 units per cheek, onabotulinum: 10 to 20 units per cheek	Three treatments with an interval of four to six weeks	Pulsed dye laser
Gaón [27]	onabotulinumtoxin A	10 ml of saline in a 100 U bottle of BoNT-A (10 units/ml)	The face’s right side: intradermal injections of 5 U at every 2 cm2. The left side: 5 U delivered using the Ecleris® electroporator	NR	Micellar cleansing lotion, moisturizing cream, and SPF 50 + sunscreen
Vasconcellos [30]	onabotulinumtoxin A	100 U vial in 1 ml of 0.9% saline solution (1 U/0.01 ml)	NR	NR	NR
Luque [38]	abobotulinumtoxinA incobotulinumtoxinA	abobotulinum: diluted to 25%, incobotulinum: diluted to 25%	abobotulinumtoxinA: A total of 30 U (0.75 U in 0.02 mL per point), with 1 cm spacing. incobotulinumtoxinA: A total of 14 U (0.25 U per 0.02 mL injected at every point), with 1 cm spacing.	NR	nr
Babadjouni [36]	NR	reconstituted with sterile saline to achieve 1.25 IU/0.1 ml	A total of 35 U of intradermal microdroplet BoNT-A over two sessions. In session one, 20 U at 0.5 cm intervals. Session two: after four weeks, with 15 U at the same dilutions, intervals, and sites.	no	Isotretinoin 20 mg daily was concomitantly started at treatment 1.
Calvisi [32]	onabotulinumtoxin A	50 units reconstituted with 0.75 ml saline & 0.5 ml lidocaine, for a total volume of 1.25 ml.	Tiny droplets (~0.01 ml, 0.2 UB) were injected in a regular grid, 1 cm2 apart. Two sessions at 14-day intervals.	NR	NR
Tong [28]	NR	100 U BoNT-A dissolved in 2.5 ml normal saline and diluted to 1 U/0.1 ml.	Experimental group: intradermal injection of BoNT-A. Control group: intradermal injection of the same amount of normal saline. 0.05–0.1 ml was injected at each point, with 0.5 cm intervals.	Three times one month apart	Broadband light
Yang [35]	NR	100 U BoNT-A was added to 6.25 ml sterile 0.9% saline for a final concentration of 16 U/ml.	0.05 ml of (0.8 U) BoNT-A solution was injected at each point, with 1 cm spacing. A total of 40–60 units of BoNT-A were used for each patient.	Some patients at five to six months	no

Assessment of the Risk of Bias in the Included Studies

The quality of the case reports was good (achieving a sum of 7/7 or 6/7 positively answered points), except for the study of Bharti et al. [37] which did not report the patient’s demographic data nor his history and manifestations at the time of presentation. They did not also report whether any adverse events occurred or not and the follow-up period after the intervention was not stated. The study by Luque et al. [38] also did not report the occurrence of adverse events (Table 4).

**Table 4:** Risk of Bias Assessment for the Included Studies Based on the Joanna Briggs Institute (JBI) Critical Appraisal Checklist for Case Reports Q1: Were patient's demographic characteristics clearly described?; Q2: Was the patient’s history clearly described and presented as a timeline?; Q3: Was the current clinical condition of the patient on presentation clearly described?; Q4: Were diagnostic tests or assessment methods and the results clearly described?; Q5: Was the intervention(s) or treatment procedure(s) clearly described?; Q6: Was the post-intervention clinical condition clearly described?; Q7: Were adverse events (harms) or unanticipated events identified and described?; Q8: Does the case report provide takeaway lessons?

Studies	Q1	Q2	Q3	Q4	Q5	Q6	Q7	Q8
Dayan [17]	Yes	Yes	Yes	NA	Yes	Yes	Yes	Yes
Park [19]	Yes	Yes	Yes	NA	Yes	Yes	Yes	Yes
Bharti [37]	No	No	No	NA	Yes	Yes	No	Yes
Luque [38]	Yes	Yes	Yes	NA	Yes	Yes	No	Yes
Babadjouni [36]	Yes	Yes	Yes	NA	Yes	Yes	Yes	Yes

As regards the other study types, all studies clearly stated the study questions and/or objectives, listed the eligibility criteria, and included patients that are representative of rosacea patients in general. However, all trials were deficient in reporting whether all eligible participants who met the pre-specified entry criteria were enrolled. Moreover, the sample size was relatively small. The details of the intervention were not clearly described in the study by Eshghi et al. [25] which did not mention the type of toxin and its dilution and the study by [30] which did not state the injected dose of the toxin. Most studies clearly defined valid and reliable outcome measures, but two studies used questionnaires for assessing patient satisfaction [29] and quality of life [27] which were not clearly described. Six studies reported that the outcome assessors were blinded to the intervention [21,26,28,31,33,35]. On the other hand, two studies were open-label [25,30], although the bias is unlikely in the study by Eshghi et al. [25] as the outcome is assessed by a patient-filled questionnaire. The remaining four studies did not provide information on the blinding of the outcome assessors [27,29,32,34]. The loss to follow-up after baseline was 20% or less in most studies [25-27,30-32,34,35], but in the study of Bloom et al. [21] as it reached 40%. Meanwhile, three studies did not provide enough information on the loss to follow-up [28,29,33]. Only three studies did not perform statistical tests [27,29,32]. Repeated measurements of the outcomes were performed in all the studies except for the study by Eshghi et al. [25]. The study by Dayan et al. [17] had a potential conflict of interest as one of the investigators was a consultant, investigator, and speaker for the pharmaceutical company that manufactured the BoNT-A. The study by Kim et al. [26] showed an added risk of bias (ROB), as a pharmaceutical provided the research funds and the used drugs. Overall, the studies with the highest ROB were those by Eshghi et al. [25] and Al-Niaimi et al. [29], followed by the studies by Gaón et al. [27], Vasconcellos et al. [30], and Calvisi et al. [32]. The quality aspects with the highest ROB included adequate sample size and blinding of outcome assessors (Table 5).

**Table 5:** Risk of Bias Assessment for the Included Studies Based on the National Institutes of Health (NIH) Quality Assessment Tool for the Before-After (Pre-Post) Study Q1: Was the study question or objective clearly stated?; Q2: 2. Were eligibility/selection criteria for the study population prespecified and clearly described?; Q3: Were the participants in the study representative of those who would be eligible for the test/service/intervention in the general or clinical population of interest?; Q4: Were all eligible participants that met the prespecified entry criteria enrolled?; Q5: Was the sample size sufficiently large to provide confidence in the findings?; Q6: Was the test/service/intervention clearly described and delivered consistently across the study population?; Q7: Were the outcome measures prespecified, clearly defined, valid, reliable, and assessed consistently across all study participants?; Q8: Were the people assessing the outcomes blinded to the participants' exposures/interventions?; Q9: Was the loss to follow-up after baseline 20% or less? Were those lost to follow-up accounted for in the analysis?; Q10: Did the statistical methods examine changes in outcome measures from before to after the intervention? Were statistical tests done that provided p values for the pre-to-post changes?; Q11: Were outcome measures of interest taken multiple times before the intervention and multiple times after the intervention (i.e., did they use an interrupted time-series design)?; Q12: If the intervention was conducted at a group level (e.g., a whole hospital, a community, etc.) did the statistical analysis take into account the use of individual-level data to determine effects at the group level? CD: cannot determine; NA: not applicable.

Studies	Q1	Q2	Q3	Q4	Q5	Q6	Q7	Q8	Q9	Q10	Q11	Q12
Bloom [21]	Yes	Yes	Yes	CD	No	Yes	Yes	Yes	No (60%)	Yes	Yes	NA
Eshghi [25]	Yes	Yes	Yes	CD	No	No (type of BoNT-A & dilution)	Yes	No (unlikely to bias)	Yes	Yes	No	NA
Silva [34]	Yes	Yes	Yes	CD	No	Yes	Yes	CD	Yes	Yes	Yes	NA
Park [31]	Yes	Yes	Yes	CD	No	Yes	Yes	Yes	Yes	Yes	Yes	NA
Friedman [33]	Yes	Yes	Yes	CD	No	Yes	Yes	Yes	CD	Yes	Yes	NA
Kim [26]	Yes	Yes	Yes	CD	No	Yes	Yes	Yes	Yes	Yes	Yes	NA
Al-Niaimi [29]	Yes	Yes	Yes	CD	No	Yes	CD	CD	CD	No	Yes	NA
Gaón [27]	Yes	Yes	Yes	CD	No	Yes	CD	CD	Yes	No	Yes	NA
Vasconcellos [30]	Yes	Yes	Yes	CD	No	No (dosing)	Yes	No	Yes	Yes	Yes	NA
Calvisi [32]	Yes	Yes	Yes	CD	No	Yes	Yes	CD	Yes	No	Yes	NA
Tong [28]	Yes	Yes	Yes	CD	No	Yes	Yes	Yes	CD	Yes	Yes	NA
Yang [35]	Yes	Yes	Yes	CD	No	Yes	Yes	Yes	Yes	Yes	Yes	NA

Results of Narrative Synthesis

All studies assessed the change in the severity of erythema/flushing after the injection of botulinum toxin, except the study by Eshghi et al. [25]. The 16 studies [17,19,21,26-38] reported a remarkable improvement in the symptoms of erythema/flushing after receiving the intervention. Different methods were used to assess the symptoms of erythema/flushing in the studies, as most studies used the Clinician Erythema Assessment (CEA) grading system. Other methods included the measurement of the erythema index (EI), the global flushing symptom score (GFSS), and the Subject Global Aesthetic Improvement Scale (sGAIS). The improvement was observed one to two weeks after the injection and persisted for a duration that ranged from three to six months, requiring repeated sessions of injection in some patients (Table 6).

**Table 6:** Summary of the Improvement in Erythema/Flushing in the Included Studies (N = 16) CEA: Clinician Erythema Assessment; EI: erythema index; GFSS: global flushing symptom score; PSA: Patients self-assessment; sGAIS: Subject Global Aesthetic Improvement Scale.

Study	Method of assessment	Reported data
Dayan [17]	assessed but methods were not reported	Decreased flushing, erythema, and inflammation within one week and persisted for three months.
Bloom [21]	standardized grading system (0=absent, 1=mild, 2=moderate, and 3=severe) on standardized digital photographs	Significant improvement in erythema scores at one, two, and three months after treatment compared to baseline. Mean difference at three months from baseline: 0.800±0.145 (p<0.001) 93% had some improvement in facial erythema. No patients suffered from worsened rosacea.
Park [19]	assessed but methods were not reported	By one week after the second treatment, a good esthetic result was achieved.
Bharti [37]	assessed but methods were not reported	Significant reduction in erythema, oedema, telangiectasias, flushing, and papulopustular lesions within one to two weeks and lasting for three to four months
Silva [34]	- CEA - CR-300® colorimeter (Konica Minolta Brazil, São Paulo, Brazil)	Improvement of the facial erythema and flushing during the following three months, with symptoms returning by the sixth month after treatment
Park [31]	- 4-point scale (0=normal, 1=mild, 2=moderate, and 3=severe) - EI by Mexameter MX18 (Courage-Khazaka electronic GmbH, Cologne, Germany).	- The mean EI decreased from 383.93±63.82 st baseline to 304.71±74.63 at eight weeks on the right cheek and from 365.4±60.42 to 308.82±83.46 on the left cheek (p<0.05). - Erythema severity score decreased from 1.9±0.27 at baseline to 1.23±0.48 at eight weeks after treatment (p<0.05). - Telangiectasia severity score decreased from 1.65±0.52 at baseline to 0.94±0.44 at eight weeks after treatment (p<0.05).
Friedman [33]	CEA (0=none, 1=almost none, 2=mild, 3=moderate, and 4=severe). EI by MX18 Mexameter (CK Electronic GmbH, Cologne, Germany). Patient self-assessment (PSA) scores (0=none, 1=almost none, 2=mild, 3=moderate, and 4=severe).	The average CEA and PSA scores at one, three, and six months significantly improved compared with baseline (P<0.001). The average Mexameter scores at baseline, one, three, and six months were 399.12, 211.18, 236.25, and 299.62 (P<0.001), respectively. The greatest effect was one month after treatment with a slight gradual recurrence of erythema at three and six months, but none returned to the initial baseline values.
Kim [26]	- CEA scale - GAIS. - EI by Mexameter	- The mean CEA score of the BoNT-A-treated side was significantly lower at weeks four and eight (p<0.01). - The mean GAIS scores of the BoNT-A-treated side were significantly higher at weeks two, four, and eight (p <0.05, <0.01, and <0.01, respectively). - EI decreased in the BoNT-A-treated side significantly at weeks four and eight as compared with baseline values (p<0.01 and <0.01, respectively) and with the normal saline-treated side (p<0.05 and <0.05, respectively).
Al-Niaimi [29]	Erythema quantification measurement using Antera 3D camera (Miravex Limited, Ireland). CEA Grading Scale (0=no erythema; 5=severe erythema)	All patients had improved erythema, telangiectasia, flushing, pruritus, and symptoms of burning sensation. Most patients sustained improvement up to nine-month follow-up with few patients having a recurrence of flushing of less severity.
Gaón [27]	Erythema quantification using Vectra® system (Canfield, Wentworth Point, Australia) with vascular programming and a red colorimetric scale.	At six weeks after the electroporation therapy, 80% improvement by 1-to-3 degrees and erythema worsened by one degree in 6.67% and no changes in 13.33% (mean improvement 1.4±1.12). At 12 weeks after needle therapy: 85.71% improvement by 1-to-3 degrees but erythema worsened by one degree in 7.14% and no changes in 7.14% (mean improvement 1.43±1.09). This effect persisted until week 12.
Vasconcellos [30]	Clinical evaluation, photographic documentation, and quantification of erythema intensity (1=absent, 2=erythema/mild flushing, 3=erythema/moderate flushing, 4=erythema/intense flushing, 5=very intense erythema/flushing	62.5% of patients reported improvement within 30 days, 12.5% improved within 90 days, and 25% did not improve. - Flushing intensity after exposure to LED light: decreased in 63% of patients, persisted in 25% and increased in 12%.
Luque [38]	assessed but methods were not reported	CASE 1: improvement of 75% of flushing and 65% of permanent erythema one month after treatment. CASE 2: 70% reduced ­flushing and erythema one month later. CASE 3: 60% reduction in erythema three weeks later.
Babadjouni [36]	assessed but methods were not reported	Significant clinical improvement and patient satisfaction were achieved.
Calvisi [32]	Subject Global Aesthetic Improvement Scale (sGAIS)	After two weeks, reduced erythema and flushing and a significant improvement in skin quality but a touch-up injection session was needed for all patients. No patients suffered from either worsening or “rebound” flaring of rosacea.
Tong [28]	GFSS (none=0; Mild=1–3; Moderate=4–6; Severe=7–9; extremely severe=10). VISIA red value EI by the multi-functional test platform MPA10 (Multi Probe Adapter) system	Compared with the control group, the GFSS, VISIA red value, and EI in the treatment group were significantly lower (p<0.05) three months after the first treatment. Compared with baseline in the experimental group: the VISIA red value (41.06±4.81 vs. 51.57±6.18), GFSS (1[0.89, 1.93] vs. 7[6.52, 7.85]), and EI (428.55±56.38 vs. 521.61±33.97) significantly decreased at six months (p<0.05).
Yang [35]	CEA scale (0=clear, 1=almost clear, 2=mild, 3=moderate, and 4=severe). Facial photographs using a VISIA Canfield imaging system (Fairfield, NJ, USA). Modified questionnaire of flushing symptoms	The CEA scores decreased from 2.88±0.62 to 1.00±0.37 at one month after treatment (P=0.000). The mean flushing scores decreased from 47.81±8.68 to 26.50±7.93 on the cheek at six months after treatment (P=000).

Eight studies [25,27,29,31-35] assessed the change in the patient’s quality of life/satisfaction after treatment with BoNT-A. Five studies [25,32-35] used the Dermatological Quality of Life Index (DLQI) questionnaire, while one study used a 5-point scale [31], another study [29] used patient satisfaction scores which were not clearly described, and the third [27] used a quality of life questionnaire which was not also clearly described. All studies reported marked improvement in the patient’s quality of life/satisfaction except for one study [34] that did not detect any statistically significant difference from the baseline (Table 7).

**Table 7:** Summary of the Improvement in Quality of Life/Patients’ Satisfaction in the Included Studies (N = 8) DLQI: the Dermatological Quality of Life Index; QoL: quality of life.

Study	Method of assessment	Reported data
Eshghi [25]	DLQI	In all patients, DLQI decreased, in two months follow-up from 8.08±1.17 to 4.5±1.21 (p<0.005).
Silva [34]	DLQI	No statistically significant differences between the months of treatment regarding the baseline.
Park [31]	5-point scale (0=very poor, 1=poor, 2=moderate, 3=good, and 4=very good)	Satisfaction ratings were 2.45±0.54, 2.79±0.71, 2.84±0.55, and 2.94±0.56 at weeks one, two, four, and eight, respectively, showing a steady increase in satisfaction.
Friedman [33]	DLQI at six months	DLQI Scores significantly improved from 18.6±1.9 at baseline to 9.6±2.8 at six months after treatment (P<0.001).
Al-Niaimi [29]	Patient satisfaction scores	Patients experienced high satisfaction with the treatment.
Gaón [27]	QoL questionnaire	More than 90% of patients had a positive impact on their quality of life.
Calvisi [32]	DLQI	Improvement of DLQI (medium improvement score >47.73).
Yang [35]	DLQI	The DLQI values decreased from 22.25±5.25 to 10.56±3.53 at six months after treatment (P=000).

Fourteen studies commented on the adverse events experienced after the injection of BoNT-A [17,19,21,25-33,35,36]. Pain at the injection site was described by most studies and was tolerable and transitory [19,21,25-28,32,33,35,36]. Erythema at the injection site was reported by six studies [26-29,33,35]. The erythema was described as transient and lasted for a few days [26,28,29]. Applying cold compresses for 20 minutes decreased the erythema [35]. Bruising at the injection site was mentioned in six studies [19,27,29,30,32,35]. The bruising spontaneously disappeared after five to 10 days. The bruising was in the form of purpura in one study [29]. Paralysis of the facial muscles was stated by four studies [28,30,31,35]. Park et al. [31] reported the occurrence of unnatural facial expressions in three patients, which improved within three months without treatment. Vasconcellos et al. [30] found one patient with a mild asymmetrical smile and treated the patient by injecting 1U of BoNT-A in the contralateral zygomaticus major. Tong et al. [28] observed a slight restriction in the lifting of the corners of the mouth in one patient 10 days after injection. The condition recovered after one month. Yang et al. [35] reported the sensation of tightness in the injection area one week after the injection in three patients, but the tightness disappeared after one month. In addition, one patient experienced a slightly asymmetrical facial expression two weeks after injection, which disappeared after one month (Table 8).

**Table 8:** Summary of the Botulinum Toxin-A-Related Adverse Effects in the Included Studies (N = 14)

Study	Pain at the injection site	Erythema at the injection site	Bruising at the injection site	Facial muscle paralysis
Dayan [17]	None	None	None	None
Bloom [21]	Transitory minimal discomfort	None	None	None
Park [19]	Mild pain during injection	None	Bruising (one patient, spontaneously disappeared after one week)	None
Eshghi [25]	Mild discomfort during the intradermal injection	None	None	None
Park [31]	None	None	None	Unnatural facial expressions (three patients) (improved within three months without treatment)
Friedman [33]	Mild discomfort	Transient erythema, oedema (self-limited)	None	None
Kim [26]	Pain during injection	Mild erythema in all patients (mean duration: 2.21 days)	None	None
Al-Niaimi [29]	None	Reactive erythema and oedema for a few days post-laser treatment	Mild transient purpura (one patient) for 10 days	None
Gaón [27]	Pain (one patient) and tingling at the application site (one patient)	temporary erythema after application (3 patients)	Ecchymosis (three patients)	None
Vasconcellos [30]	None	None	Ecchymosis (one patient, resolution within 5 days)	Mild asymmetrical smile (one patient, treated with 1U of BoNT-A in contralateral zygomaticus major)
Babadjouni [36]	Mild, localized pain at the injection site during the procedure	None	None	None
Calvisi [32]	Mild pain during the treatment	None	Bruising (spontaneously disappeared after one week)	None
Tong [28]	The pain during injection was tolerable and comparable between the two sides	Mild erythema after injection (all patients, mean duration: 2.05±0.72 days).	None	Slightly restricted lifting of the corners of mouth 10 days after injection (one patient), recovered after one month
Yang [35]	Slight pain during injection (all patients), but the pain was transient and tolerable.	Erythema (four cases, decreased after 20 min of cold compress).	Bruises (five cases, subsided after one week)	Tightness in the injection area one week after injection (three patients) but disappeared after one month. slightly asymmetric facial expression at two weeks after injection (one patient), disappeared after one month.

Pooling of the Incidence Rates of the Adverse Events

After excluding the case reports, we performed a pooling of the incidence rates of the reported adverse events in the studies which reported the number of patients experiencing the adverse event. We were unable to pool the rate of pain at the injection site as the number of patients was not exactly reported in most studies, probably because it was a common complaint in most patients. There was considerable heterogeneity in the reported rates of localized erythema (Q = 200.130, p<0.001, I2=96%) and ecchymosis (Q = 19.152, p = 0.024, I2=53%), so the random effects model was used for pooling the incidence. As for the affection of facial muscles, the heterogeneity was not significant (Q = 13.288, p = 0.208, I2=24.7%), so the fixed effect model was used (Table 9).

**Table 9:** Pooling of the Incidence of the Reported Adverse Events CI: confidence interval; Q: Chocran’s Q test; * significant at p<0.1

Adverse effects	Studies, N (patients)	Model	Incidence	95% CI	Q	p-value	I^2 ^(%)
Localized erythema	9 (145)	Random	24.6%	0 – 65.8%	200.130	<0.001*	96.0
Localized ecchymosis	10 (166)	Random	5.1%	1.1 – 11.3%	19.152	0.024*	53.0
Affection of facial muscles	11 (181)	Fixed	4.3%	1.8 – 7.8%	13.288	0.208	24.7

**Figure 2:**
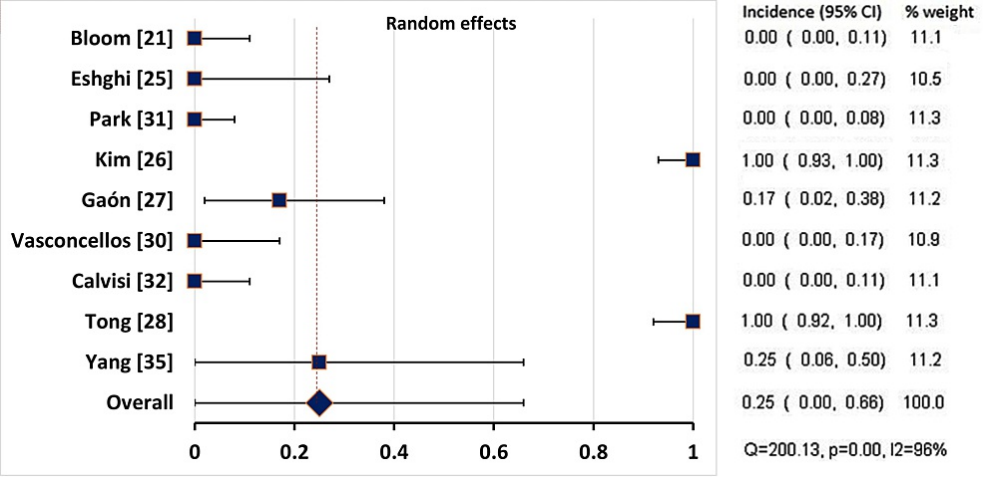
Forest Plot Showing the Incidence of Localised Post-Injection Erythema in all Studies CI: confidence interval

The pooled rate of localized erythema was 24.6% (95% CI: 0 - 65.8%), based on the results of nine studies (145 patients) (Table 9, Figure 2).

**Figure 3:**
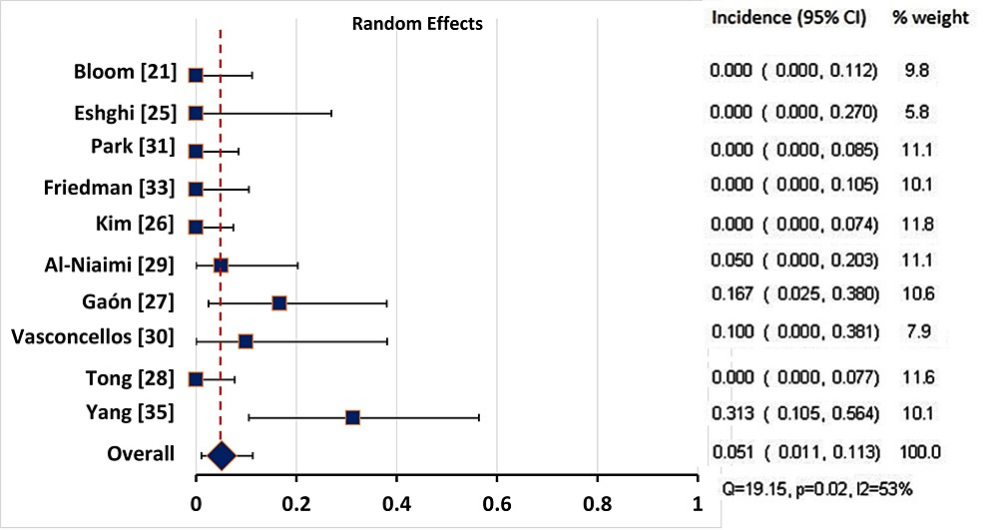
Forest Plot Showing the Incidence of Localised Post-Injection Ecchymosis in all Studies CI: confidence interval

The pooled rate of localized ecchymosis was 5.1% (95% CI: 1.1 - 11.3%), based on the results of 10 studies (166 patients) (Table 9, Figure 3).

**Figure 4:**
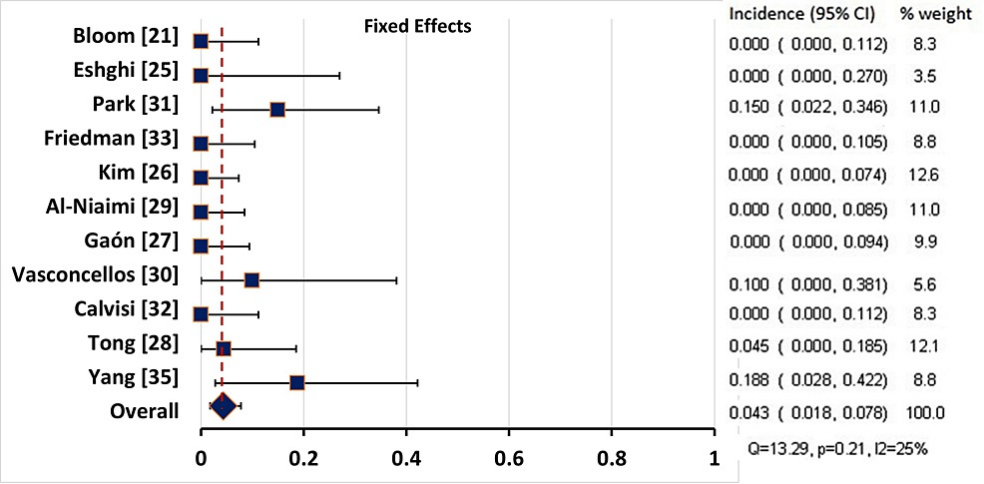
Forest Plot Showing the Incidence of Affection of Facial Muscle Movements in all Studies CI: confidence interval

The pooled incidence of facial muscle affection was 4.3% (95% CI: 1.8 - 7.8%), based on the results of 11 studies (181 patients) (Table 9, Figure 4).

Discussion

Summary of the Main Findings

Several treatment modalities have been advocated for the treatment of rosacea, including topical, oral, laser-based, light-based, and injection therapies. These treatments can also be used in combination. The therapeutic effect of the treatments is based on their action against demodex, inflammatory mediators, and/or angiogenesis [39,40]. These modalities are effective in many rosacea patients, but some cases are refractory to conventional therapies and represent a challenge for treatment [41].

Several case reports and case series have reported that intradermal BoNT-A improved flushing and telangiectasia in rosacea patients [17-21]. However, the use of BoNT-A in treating rosacea is still off-label. The evidence of the efficacy and safety of BoNT-A in rosacea patients is still not established. Consequently, current clinical guidelines do not recommend its routine use in treating rosacea. Therefore, the present systematic review was carried out to summarize the evidence regarding the efficacy and safety of BoNT-A in the treatment of patients with rosacea.

Seventeen studies were retrieved using the search strategy in this systematic review [17,19,21,25-38]. The studies showed wide variations in design, the dosing regimen of BoNT-A, and the methods used to assess the outcomes.

The results of the included studies showed that BoNT-A was effective in reducing the severity of erythema and flushing in rosacea patients. The improvement was noticed one to two weeks post-injection and the effect persisted for three to six months. By this time, some patients showed recurring symptoms of rosacea (though with less intensity than at baseline) requiring repeated sessions of injection. We were unable to assess the rate of recurrence after the first injection as the studies did not provide the exact numbers of patients with recurred symptoms.

The improvement of the embarrassing symptoms of rosacea seemed to impact favourably the patients’ quality of life [25,27,29,31-33,35]. but one study [34] reported the lack of any statistically significant difference from the baseline, which may be attributed to the small sample size of the study.

The mechanism of action of BoNT-A is presumably due to blocking the release of acetylcholine from peripheral nerves, thereby reducing vasodilatation of the cutaneous blood vessels [42,43]. Other neurotransmitters were also suggested that could be reduced by BoNT-A injection, including substance P, calcitonin gene-related peptide, and glutamate [44].

The use of BoNT-A seems to have a good safety profile, as no serious adverse events (such as anaphylaxis) were reported by any of the studies. The most common adverse event reported after BoNT-A injection was localized pain at the injection site which was tolerable and transient. Post-injection erythema was observed in some studies [26-29,33,35], which was transient and resolved within a few days. Localized post-injection ecchymosis was also reported to occur in some patients, lasting five to 10 days [19,27,29,30,32,35]. All these adverse events were tolerable and temporary, requiring no treatment in most cases. The pooled rates of localized erythema and ecchymosis were 24.6% (95% CI: 0 - 65.8%) and 5.1% (95% CI: 1.1 - 11.3%), respectively.

The most feared adverse event in using BoNT-A is paralysis of the facial muscles, but this complication was reported only by four studies [28,30,31,35]. In three studies, motor affection resolved spontaneously within a few months [28,31,35]. In one study, the patient was treated by injecting 1U of BoNT-A in the contralateral zygomaticus major to adjust for a mild asymmetrical smile [30]. The pooled incidence rate of facial muscle affection was 4.3% (95% CI: 1.8 - 7.8%).

The studies used different types of BoNT-A, the most common was onabotulinumtoxinA [17,19,27,29-32], followed by abobotulinumtoxinA [21,29,33,38], while the least used were incobotulinumtoxinA [38] and prabotulinumtoxinA [26]. Six studies did not mention the exact type of BoNT-A [25,28,34-37]. Previous evidence from the literature suggests that abobotulinumtoxinA could more easily diffuse and migrate through the skin, which helps the spread of the injected toxin if facial flushing covers large areas [45,46].

The BoNT-A is characterized by high molecular weight, so its penetration of the intact stratum corneum is poor [47]. Thus, the toxin is typically administered by intradermal injection. Disruption of the stratum corneum can increase the skin permeability, allowing for topical application of BoNT-A instead of the injection technique [48]. Ablation of stratum corneum can be done using thermal methods [33] or physical approaches [27]. All the studies used the intradermal injection of BoNT-A, but two studies assessed other methods of drug delivery. Friedman et al. [33] used a novel non-laser thermal resurfacing system (Tixel; Novoxel, Netanya, Israel), and the results showed good efficacy without suffering motor paralysis. Gaón et al. [27] used facial electroporation, which is a technique that exposes the skin to a light electric field, reducing the resistance of the cell wall, with comparable results to the intradermal injection. These alternative methods of drug delivery can reduce the pain that occurs with intradermal injection, which was observed in the study by Gaón et al. [27]. Also, these alternative techniques can reduce the rate of facial motor paralysis which occurrence is related partially to the depth of injection.

Overall Completeness, Applicability, and Quality of the Evidence

The present systematic review attempted to summarize the current evidence on the efficacy and safety of BoNT-A as a treatment for rosacea. The results of the systematic review show that BoNT-A is an effective and safe treatment for alleviating the symptoms of rosacea and improving the patient’s quality of life. However, included studies showed several limitations that require caution before recommending the routine use of BoNT-A. The included studies have small sample sizes and none of them performed a justification for the sample size. Besides, there are concerns regarding the methodological quality of some studies. These concerns include selection bias as the number of eligible patients who were not enrolled was not stated by any of the studies. In addition, performance bias was raised as two studies did not describe the used questionnaires for assessing patient satisfaction [29] and quality of life [27].

Attrition bias was a concern in four studies: one in which the loss to follow-up was 40% [21] and three studies which did not report the patients' numbers at the time of measuring the outcomes [28,29,33]; a considerable loss to follow-up implies that patients may have withdrawn due to inefficacy or intolerable side effects. Moreover, even in patients who completed the follow-up, the duration was not adequate to ensure that delayed adverse effects would not develop. Only six studies reported the blinding of the outcome assessors, thus detection bias is a possibility in the remaining studies.

Furthermore, the heterogeneous reporting of the measured outcomes by the studies made the performance of meta-analysis for efficacy unfeasible. Pooling of the incidence rates of the reported adverse events was done. Meanwhile, if the reporting of the outcomes was standardized across most studies, a meta-analysis could have been performed to elucidate better the efficacy of BoNT-A in relieving the symptoms of erythema and flushing.

A previous systematic review assessed the evidence for the use of BoNT-A in rosacea patients [41], but it included only eight of the studies assessed in the present systematic review. The systematic review similarly concluded that BoNT-A potentially has a satisfactory efficacy and safety profile as a treatment for rosacea.

The limitations of the available studies call for the conduction of future large-scale clinical trials to confirm the effectiveness and define the optimal dosing regimen and the rate of recurrence. Future studies should ensure the blinding of the outcome assessors and allow for an adequate follow-up after the treatment, with repeated measurements of the outcomes.

## Conclusions

BoNT-A seems to be effective in alleviating the erythema and flushing of rosacea as well as increasing patients’ satisfaction or improving their QoL. The rates of adverse events were relatively low, which included localized erythema, localized ecchymosis, and facial muscle affection. The recurrence of the symptoms a few months after the injection requires repeated sessions, which may raise cost-effectiveness issues. The currently available studies have several weaknesses, including small sample sizes, risk of selection bias, non-description of questionnaires used to assess patient satisfaction and quality of life, and non-reporting or presence of large numbers of loss to follow-up.
